# Understanding why edema in salvaged myocardium is difficult to detect by late gadolinium enhancement

**DOI:** 10.1186/1532-429X-14-S1-O63

**Published:** 2012-02-01

**Authors:** Martin Ugander, Paul S Bagi, Julian O Booker, Li-Yueh Hsu, Abiola J Oki, Andreas Greiser, Peter Kellman, Anthony H Aletras, Andrew E Arai

**Affiliations:** 1 National Heart, Lung and Blood Institute, National Institutes of Health, Bethesda, MD, USA; 2 Dept of Clinical Physiology, Karolinska Institute, Stockholm, Sweden; 3 Siemens AG Healthcare Sector, Erlangen, Germany

## Background

T2-weighted cardiac magnetic resonance (CMR) can visualize myocardial edema in salvaged myocardium which appears non-infarcted by late gadolinium enhancement (LGE) CMR. However, the mechanisms governing why LGE does not visualize edema in non-infarcted myocardium remain unclear. The objective of the study was to evaluate the extracellular volume fraction (ECV) of edematous salvaged myocardium using quantitative T1-mapping techniques in order to better understand why this tissue may be difficult to detect by LGE imaging.

## Methods

Dogs (n=10) underwent coronary occlusion and reperfusion, followed by 1.5T CMR. Salvaged myocardium was defined as having bright signal intensity on T2-prepared steady-state free precession (T2-prep) images and the absence of infarction by LGE, and signal intensities were quantified SD units brighter than remote myocardium. Myocardial extracellular volume fraction (ECV) was measured by T1 quantification before and after Gd-DTPA contrast administration and calibration by blood hematocrit.

## Results

LGE signal intensity of salvaged and infarcted myocardium were 1.7±0.4 and 8.1±1.5 SD from remote, respectively. T2-prep signal intensity of salvaged and infarcted myocardium were 2.8±0.2 and 4.9±1.0 SD from remote, respectively. Compared to remote myocardium, T1 of salvaged myocardium was 14% higher before contrast (1050±114ms vs 919±66ms, p<0.001) and 10% lower 30 minutes after contrast (461±57ms vs 512±67ms, p<0.001). The ECV of salvaged myocardium was 34+/-7% which was significantly different than ECV of normal myocardium 24+/-3% (p=0.04).

## Conclusions

Salvaged myocardium has a post-contrast T1 which is approximately 50 ms less than remote myocardium and has an LGE image intensity less than 2 SD from remote, making it difficult to appreciate in LGE images. However, contrast concentration, and thus extracellular space, is determined by the change in 1/T1 (R1) which occurs from pre- to post-contrast. The combined and directionally opposed differences in pre- and post-contrast T1 explain why the extracellular volume fraction of salvaged myocardium is increased compared to remote. LGE image intensity does not incorporate information on pre-contrast T1, thus giving the impression of no change in the size of the extracellular space of salvaged myocardium despite the presence of extracellular edema.

## Funding

This work was supported by the Intramural Research Program of the National Heart, Lung, and Blood Institute, National Institutes of Health, USA [1 Z01 HL004607-08 CE].

**Figure 1:**
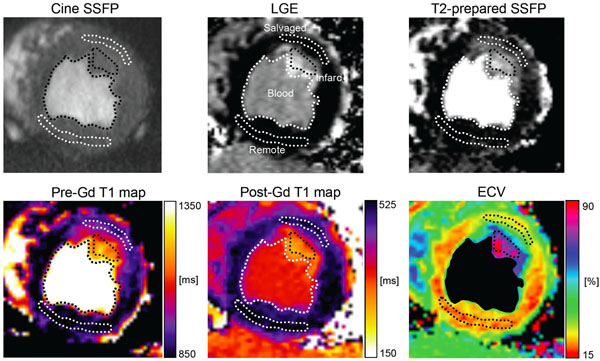
A representative short-axis slice from a dog that has undergone coronary occlusion and reperfusion. Blood pool, remote myocardium, infarcted myocardium and salvaged myocardium are delineated in the respective images. Note how salvaged myocardium is hypointense on LGE (indicating no infarction), hyperintense on T2-prepared SSFP (indicating edema), has an increased pre-gadolinium T1, barely perceivable reduced post-gadolinium T1, and an increased extracellular volume fraction (ECV). The blood pool of the ECV image has been set to black for clarity.

